# The Medical and Surgical Reporter

**Published:** 1859-03

**Authors:** 


THE MEDICAL AND SURGICAL REPORTER, on the first of the year, appeared under a new guise. Dr. W. B. Atkinson having ceased his connection with it, Dr. R. J. Levis, who is well known to the profession, has become associated with Dr. Butler in the editorship of the journal, which, we are happy to see, has very much improved, both in regard to its contents and its appearance.

